# *Andrographis paniculata* aqueous extract exhibits cardioprotective effect against dichlorvos-induced toxicity, a commonly used organophosphate pesticide.

**DOI:** 10.1016/j.toxrep.2025.102038

**Published:** 2025-04-24

**Authors:** Waidi Adeoye Saka, Olusanjo Ayandiji Ayandele, Oladapo Oluwasegun Oladipo, Olamilekan Sultan Adeshina, Busuyi David Kehinde

**Affiliations:** a Department of Physiology, Faculty of Basic Medical Science, Ladoke Akintola University of Technology, Ogbomoso, Oyo State, Nigeria; b Department of Biochemistry, Faculty of Basic Medical Science, Ladoke Akintola University of Technology, Ogbomoso, Oyo State, Nigeria

**Keywords:** Andrographis paniculata, Cardiotoxicity, Dichlorvos, Cardiac markers, Oxidative stress

## Abstract

Traditional medicine is the primary healthcare source for most people in developing nations, with herbal remedies used for disorders of metabolism. The study assessed how *Andrographis paniculata* aqueous extract affected male Wistar rats' cardiotoxicity caused by dichlorvos. Three groups consisting of eighteen rats were randomly assigned (n = 6). Group A was not exposed to dichlorvos, as it served as (control). Group B was exposed to dichlorvos (1 ml/day for 1 week) via inhalation. Group C was exposed to dichlorvos (as in B) and treated with *Andrographis paniculata* aqueous extract (500 mg/kg body weight) orally for 28 days. Exposure to dichlorvos caused alteration in cardiovascular variables and electrocardiac function (blood pressure, heart rate and electrocardiogram), cardiac injury (lactate dehydrogenase and creatine kinase), oxidative stress (malondialdehyde (MDA), superoxide dismutase (SOD), reduced glutathione (GSH) and glutathione peroxidase (GPx)), cardiac inflammation (tumor necrosis factor-α (TNF-α), interleukin-6 (IL-6) and apoptosis (caspase 3). However, treatment with *Andrographis paniculata* aqueous extract improved the antioxidant defense system, attenuated electrocardiac impairment, and prevented damage to cardiac musculature. *Andrographis paniculata* aqueous extract exhibited cardioprotective potential**.**

## Highlights

• Dichlorvos-induced cardiotoxicity via oxidative stress, inflammation, and apoptosis.
• Aqueous extract of *A. paniculata* confers cardioprotection against DDVP-induced cardiotoxicity by restoring cardiac hemodynamic and enhancing endogenous antioxidant defense.
• The extract’s anti-inflammatory and anti-apoptotic properties reduce tissue injury and inflammation.
• The study also showed that whole herb extract might be used for prophylaxis and treatment of cardiovascular diseases.

## Graphical Abstract

**Figure:**
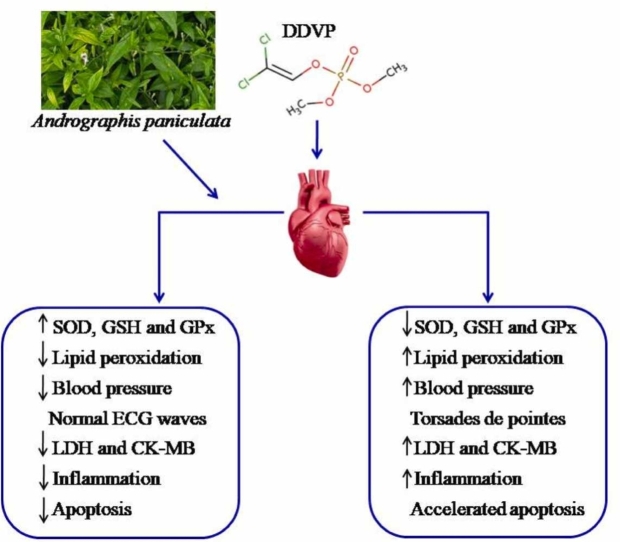

## 1 Introduction

Cardiovascular disease (CVD) accounts for around 20 percent of all fatalities globally and is a leading cause of sickness and mortality. According to estimates from the World Health Organization (WHO), almost 30 million people will die from CVD. Most CVD-related deaths occur in developing countries, predominately among people under the age of 70 [68], [73]. CVD mainly result because vascular dysfunction has the potential to harm organs. The primary factors that cause high blood pressure, thrombosis, and atherosclerosis are examples of vascular dysfunctions. Furthermore, smoking, an unhealthy diet, diabetes mellitus, hyperlipidemia, dyslipidemia, physical inactivity, high low-density lipoprotein cholesterol (LDL), low high-density lipoprotein cholesterol (HDL), and hypertension are major risk factors that significantly contribute to cardiovascular health risks [34], [67]. Apart from the fundamental risk factors, exposure to environmental toxic substances such as pesticides can also contribute to cardiovascular diseases through inflammation and oxidative stress. Consequently, ecological toxicants must be regarded as important CVD risk factors [60].

An international challenge is the use of organophosphates (OPs) in the control of key disease-carrying vectors. OPs have been connected to health risks, human poisoning, and environmental contamination [38]. Toxicity and non-specific multi-organ toxicity have been seen to rise with the release of OPs into the environment through unintentional exposure to them [39]. Dichlorvos (DDVP) is an OP pesticide that is known to have cardiotoxic effects. The production of reactive free radicals, which is probably the cause of DDVP-mediated cell damage, is one of the possible causes of the drug's negative effects [23], or abnormal calcium signaling, which could lead to irregular arrhythmia, chronic heart failure, and electrical conduction in the heart [5]. Research has shown that besides the established primary mechanism of action, acetylcholinesterase inhibition, DDVP induces oxidative stress and reduces antioxidant activities [25], [43], [50], [51].

Often referred to as the King of Bitters, *Andrographis paniculata* is a herbaceous plant of the Acanthaceae family. For centuries, people have utilized it traditionally to treat a wide range of ailments on the continents of Asia, America, and Africa. These ailments include cancer, diabetes, high blood pressure, ulcers, leprosy, bronchitis, skin diseases, flatulence, colic, influenza, dysentery, dyspepsia, and malaria [29]. The plant is also used traditionally for detoxification in China [19], [58]. It has been demonstrated that the plant's extract and pure components have antibacterial properties [1], [72], immunostimulant, immunomodulatory [46], anti-inflammatory and antioxidant [35], [40], [42], [74], anti-diabetic [32], [41], [48], [59], hepato-renal protective [42], [59], modulation of sexual function and sex hormone [40], glucose regulation and insulin sensitivity [52].

There is little information available on DDVP's effects on cardiovascular function after inhalational exposure, which is the most common route of exposure. Additionally, it is unknown how *Andrographis paniculata*, an antioxidant, may affect DDVP-induced cardiovascular changes. Consequently, it's critical to look at how an aqueous extract of *Andrographis paniculata* affects the cardiovascular alterations in male Wistar rats given dichlorvos. The purpose of this work is to determine how this extract affects the cardiovascular changes caused by DDVP in adult male Wistar rats.

## 2 Materials and methods

### 2.1 Animals

Eighteen male Wistar rats in total, about 8–10weeks old and with body weights ranging from 200 g to 250 g, used in this investigation were procured and housed at the Department of Physiology's animal house of the Ladoke Akintola University of Technology (LAUTECH), Ogbomoso. The rats were given a regular pellet diet and unlimited access to water in a well-ventilated plastic cage. The rats were acclimated to the laboratory conditions (temperature 25ºC and environment 12 h light-dark cycle) for two weeks. Faculty of Basic Medical Sciences ethics committee of LAUTECH, Ogbomoso gave approval for this study (FBMS/AEC/P/074/22). A conscious effort was made to guarantee that the fewest possible animals were used and that their suffering was kept to a minimum.

### 2.2 Treatments

The eighteen (18) rats were subjected to a random sampling technique to allocate the rats into three groups. Each group consists of six (6) rats.

♦ Group A (control): were given standard rat feed, water, and 1 ml of distilled water daily.
♦ Group B (DDVP): The rats were given standard rat feed and water and exposed to 98.4 g/m^3^ DDVP via inhalation for 15 min daily [49], [51] and 1 ml of distilled water daily through the oropharyngeal cannula. 1 ml) of DDVP was dropped on a cotton wool which was placed in a compartment of the desiccator while the rat was placed in another compartment for 15 min. There was easy diffusion of the DDVP across the desiccator, but the rat had no physical contact with the cotton wool.
♦ Group C (DDVP + *A. paniculata*): The rats were given standard rat feed and water and exposed to DDVP as described above for group B and 500 mg/kg of aqueous extract of *A. paniculate* [53] once per day through the oropharyngeal cannula for 28 days.

DDVP was administered at a dosage of 98.54 g/m3 – similar to previous studies [49], [50]. The route of administration was inhalation, as described by Saka and colleagues [49]. The dose of *A. paniculata* used is as reported in a previous study [53]. Rats were treated in accordance with the National Institute of Health Guide for Care and Use of Laboratory animals, and the study received approval from the ethical committee of the Faculty of Basic Medical Science at Ladoke Akintola University of Technology (LAUTECH), Ogbomoso.

### 2.3 Chemicals

The DDVP containing 1000 g of DDVP/Litre) used for the study was manufactured by Forward (Beinaj) Hepu Pesticide Co. Limited, China, and procured from Saro Agrosciences Limited, Oyo State, Nigeria. Standard ELISA kits were utilized for the detection of lactate dehydrogenase (LDH), creatinine kinase (CK), tumor necrosis factor-α (TNF-α), and interleukin-6 (IL-6) (Agappe Diagnostics, Switzerland).

### 2.4 Plant collection, extraction, and preparation

*Andrographis. paniculata* fresh leaves were obtained from herbarium of the Department of Pure and Applied Biology, LAUTECH, Ogbomoso with voucher number LHO 715. After air drying, the leaves were pulverized in an electric blender. In a conical flask, 300 g of *A. paniculata* powder and 3 L of distilled water were combined. The mixture was stirred multiple times, covered, and left at room temperature overnight. The next day, Whatman filter paper (15 cm) was utilized to filter the mixture, and the filtrate was evaporated at 40°C until completely dry. This resulted in a fine, dark-colored solid residue, which was scraped, weighed, and stored in a capped bottle. A fresh solution was made from the dried residue daily [37].

### 2.5 Sample collection and preparation

The animals were weighed at the start of the trial, weekly and at the end of the experiment. The difference between the weight at the start and finish of the experiment was used to calculate body weight growth. After all administration is complete (end of experimental period), the rats were anesthetized with 1 % chloralose and 25 % urethane given intraperitoneally. Blood pressure measurement and electrocardiography were carried out. After a heart puncture, blood was collected and centrifuged for five minutes at 3000 r.p.m, after which serum was collected for biochemical analysis. The hearts were excised, divided, fixed in buffered formalin, and processed for immunohistochemical staining.

### 2.6 Biochemical assays

ELISA kits were utilized to measure the serum levels of interleukin-6 (IL-6) and tumor necrosis factor-α (TNF-α) (Agappe Diagnostics, Switzerland) following the manufacturer's instructions.

Colorimetric techniques were used to measure the activities of superoxide dismutase (SOD), reduced glutathione (GSH), glutathione peroxidase (GPx), malondialdehyde (MDA), and myeloperoxidase (MPO) [6], [7], [8].

ELISA kits were used to measure the levels of lactate dehydrogenase (LDH) and creatinine kinase (CK) in serum (Agappe Diagnostics, Switzerland) following the manufacturer's instructions.

### 2.7 Cardiovascular parameters (Blood pressure, heart rate, and electrocardiography)

After the period of the administration, rats were given anesthesia. with 1 % chloralose and 25 % urethane given intraperitoneally. Anesthesia was confirmed by checking for a lack of pedal reflex. The rats were then placed on a board, and conductive gel was applied to the chest, left arm, left leg, right arm, and right leg, followed by the attachment of EDAN electrodes. The electrodes were then connected to the laptop, and information about the rats was stored there. An electrocardiogram was recorded for each rat for one minute, following a procedure described in previous studies [12]. Following the anesthesia, the rats' femoral artery was cannulated, and a pressure transducer (P23LD Statham Hato Rey, Inc.) of the Grass 7D polygraph (Grass Instrument Ltd, Quincy, Massachusetts, USA) was used to measure their blood pressure. Alongside blood pressure, heart rate was also recorded. This procedure was carried out following previously reported studies [13]. Calculating the mean arterial blood pressure was done as:

MAP = DBP + 1/3 (SBP – DBP)

MAP – Mean arterial blood pressure

SBP – Systolic blood pressure

DBP – Diastolic blood pressure.

### 2.8 Caspase-3 immunohistochemistry

Immunohistochemistry was carried out as described in previous studies [21]. Before being deparaffinized and having the antigen extracted, paraffin slices (5 μm) were dried for an hour at 37°C. The endogenous peroxidase activity was inhibited by incubating the sections for 10–15 minutes. The slides were subjected to a one-hour incubation process with the polyclonal antibody at 37°C, followed by washing and incubation with prediluted biotinylated goat antirabbit IgGs for half an hour and streptavidin horseradish-peroxidase.

$$\frac{Total\;number\;of\;caspase\;-3-positive,activated\;cells\times \;100}{Total\;number\;of\;nuclei}\;$$

The caspase-3 labeling index was calculated as the percentage of apoptotic cells per total number of cells:

### 2.9 Phytochemical analysis

Qualitative phytochemical screening of *A. paniculata* aqueous extract was done and reported as previously documented by [36].

### 2.10 Test for alkaloids

One milliliter (1 ml) of diluted hydrochloric acid (HCl) was mixed with a little amount of the extract before it was filtered. The reagent Dragandroff's was applied to the filtrate. The presence of alkaloids is indicated by the formation of organic precipitate.

### 2.11 Test for tannins

In a test tube, a little amount of the dried powder of *Andrographis paniculata* was boiled in 20 milliliters of water and then filtered. After adding a few drops of 0.1 percent ferric chloride, the presence of tannins was indicated by a blackish-blue or brownish-green tint.

### 2.12 Test for saponins

In a water bath, two (2) g of powdered *Andrographis paniculata* was boiled in 20 ml of water and then filtered. To verify if saponins are present, combine 10 milliliters of the filtrate with 5 milliliters of water in a test tube, shake well, and watch for the development of a stable foam.

### 2.13 Test for flavonoids

The extracts turned yellow when strong sulfuric acid was added, signifying the presence of flavonoids.

### 2.14 Test for terpenoids (Salkowski test)

5 g of extract were mixed with 2 milliliters of chloroform. After that, 3 ml of concentrated H_2_SO_4_ was added to create a layer. A thin interface developed a reddish-brown coloring, signifying a positive terpenoids test.

### 2.15 Test for protein

To perform Xanthoprotein tests, a small amount of extract was dissolved in 5 ml of water followed by addition of 1 ml concentrated nitric acid and stirred. As a result, a white precipitate was formed. The solution was heated for 1 minute and then allowed to cool under running water. It was made alkaline by an excess of NaOH. The appearance of an orange precipitate indicates the presence of protein.

### 2.16 Test for phytosterol

The Salkowski test was used to determine if phytosterol was present. After adding 1 ml of concentrated sulfuric acid to 1 g of plant extract, the mixture was let to stand for 5 minutes. Following shaking, the lower layer had a golden-yellow hue, which suggested the presence of phytosterol.

### 2.17 Test for glycosides

Fehling's test was performed on a little amount of the extract after it had been hydrolyzed for a few hours in a water bath with 5 ml HCL. Reducing sugars were present when a yellow or red-colored precipitate appeared.

### 2.18 Statistical analysis

In order to compare the mean values of the variables between the groups, the data were analyzed using GraphPad Prism for Windows' one-way analysis of variance (ANOVA) and Tukey's posthoc test for multiple comparisons to determine the significance of individual variation between two groups (Versions 5, GraphPad Software, Inc.). P values < 0.05 were deemed statistically significant. The data are shown as mean ± SEM.

## 3 Results

### 3.1 Estimation of body weight

**Fig. 1:**
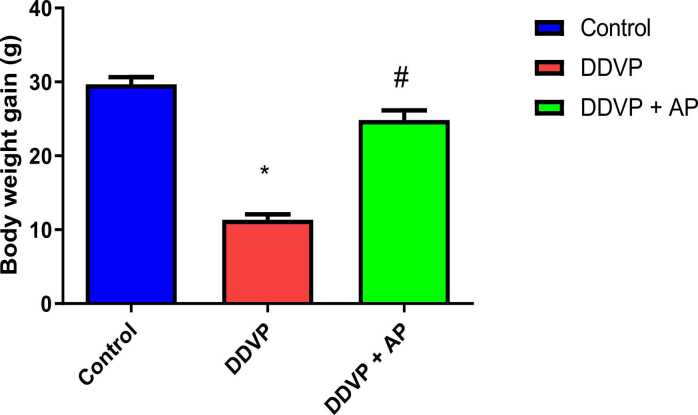
Body weight gain in control, DDVP-treated and DDVP + AP rats. The mean ± standard error of the mean (S.E.M) is shown by each bar. *p < 0.05 in comparison to the control, ^#^p < 0.05 vs DDVP-treated. DDVP: Dichlorvos; AP: *Andrographis paniculata.* Body weight is expressed in grams. Dosage of DDVP= 98.54 g/m3; Dosage of AP= 500 mg/kg.

After receiving DDVP administration, there was a significant decrease in body weight gain. On the other hand, oral administration of *A. paniculata* prevented this effect (Fig. 1).

### 3.2 Estimation of cardiovascular variables

#### 3.2.1 Blood pressure (SBP and DBP) and heart rate (HR) of rats

**Fig. 2:**
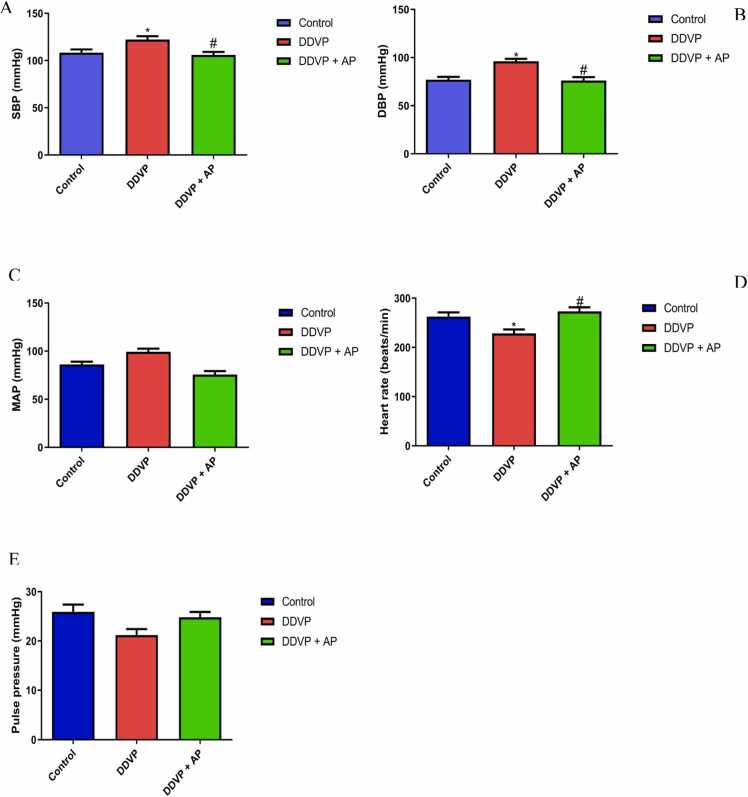
A- Systolic blood pressure; B- Diastolic blood pressure; C- Mean arterial blood pressure; D- Heart rate; and E- Pulse pressure in control, DDVP-treated and DDVP + AP treated rats. Each bar represents mean ± standard error of mean (S.E.M). *p < 0.05 vs control, ^#^p < 0.05 vs DDVP-treated. Dosage of DDVP= 98.54 g/m3; Dosage of AP= 500 mg/kg DDVP: Dichlorvos; AP: *Andrographispaniculata.* Blood pressures are expressed in mmHg and heart rate is expressed in beats/min.

Fig. 2 shows the effect of DDVP inhalation and treatment with *A. paniculata* on blood pressure parameters. DDVP increased SBP, DBP, and MAP but reduced HR when compared with the control. Treatment with *A. paniculata* attenuated DDVP-induced alterations.

### 3.3 Estimation of electro-cardiac function

#### 3.3.1 P-Duration, PR-Intervals, QRS complex, and QT-Intervals of rats

**Fig. 3:**
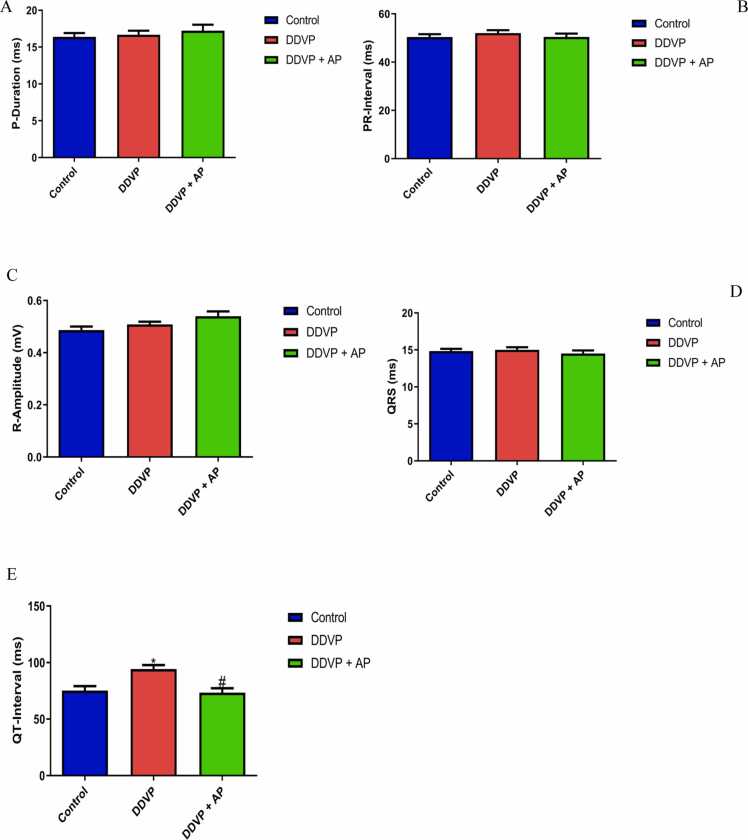
A- P duration; B- PR interval; C- R amplitude; D- QRS complex; and E- QT interval in control, DDVP-treated and DDVP + AP rats. Each bar represents mean ± standard error of mean (S.E.M). *p < 0.05 vs control, ^#^p < 0.05 vs DDVP-treated. Dosage of DDVP= 98.54 g/m3; Dosage of AP= 500 mg/kg. DDVP= Dichlorvos; AP: *Andrographis paniculata.* P duration, PR interval, QRS complex and QT interval are expressed in ms. R amplitude is expressed in mV.

The effects of DDVP and *A. paniculata* on the electrocardiogram in treated rats are shown in Fig. 3. P-duration had no significant difference. PR-interval, QRS complex, and R amplitude. However, DDVP treatment caused a significant increase in QT-interval while treatment with *A. paniculata* prevented the prolongation of QT-interval.

### 3.4 Estimation of cardiac enzymes

#### 3.4.1 Lactate dehydrogenase (LDH) and creatine kinase enzyme (CK-MB) of rats

**Fig. 4:**
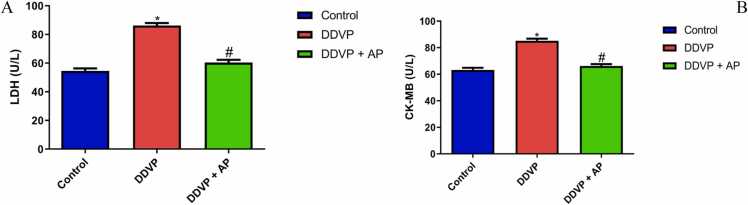
A- Lactate dehydrogenase (LDH); B- Creatine kinase (CK-MB)in control, DDVP-treated and DDVP + AP rats. Each bar represents mean ± standard error of mean (S.E.M). *p < 0.05 vs control, ^#^p < 0.05 vs DDVP-treated. Dosage of DDVP= 98.54 g/m^3^; Dosage of AP= 500 mg/kg. DDVP: Dichlorvos; AP: *Andrographis paniculata.* LDH and CK-MB are expressed in U/L.

The results of DDVP and *A. paniculata* treatment are illustrated in Fig. 4. DDVP significantly increased the concentration of LDH and CK-MB compared with the control. *A. paniculata* treatment decreased the concentration of LDH and CK-MB.

#### 3.4.2 Estimation of cardiac oxidative markers and antioxidant enzymes

**Fig. 5:**
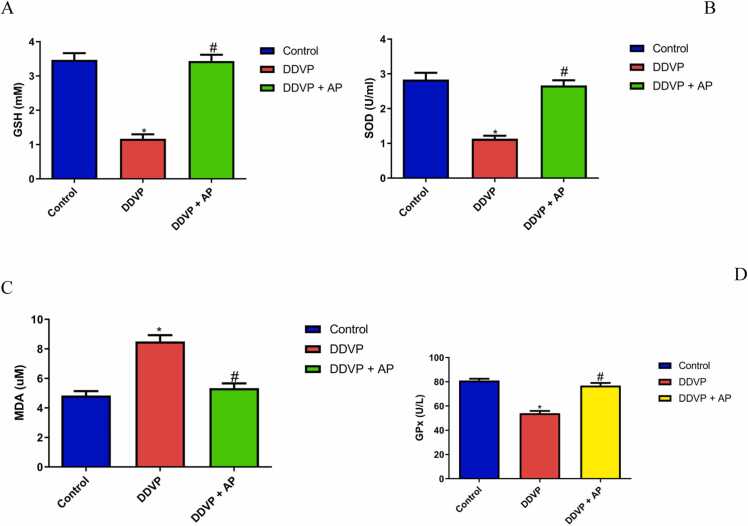
A- Reduced glutathione (GSH); B- Superoxide dismutase (SOD); C- Malondialdehyde (MDA); D- Glutathione peroxidase (GPx)in control, DDVP-treated and DDVP + AP rats. Each bar represents mean ± standard error of mean (S.E.M). *p < 0.05 vs control, ^#^p < 0.05 vs DDVP-treated. Dosage of DDVP= 98.54 g/m3; Dosage of AP= 500 mg/kg DDVP: Dichlorvos; AP: *Andrographispaniculata.*

Fig. 5 shows the results of exposure to DDVP and *A. paniculata* on lipid peroxidation and antioxidant activities. Inhalation of DDVP upregulated lipid peroxidation and diminished activities of antioxidants when compared to control rats. Oral administration of *A. paniculata* prevented lipid peroxidation and enhanced antioxidant activities.

Estimation of Cardiac inflammatory markers and apoptosis

**Fig. 6:**
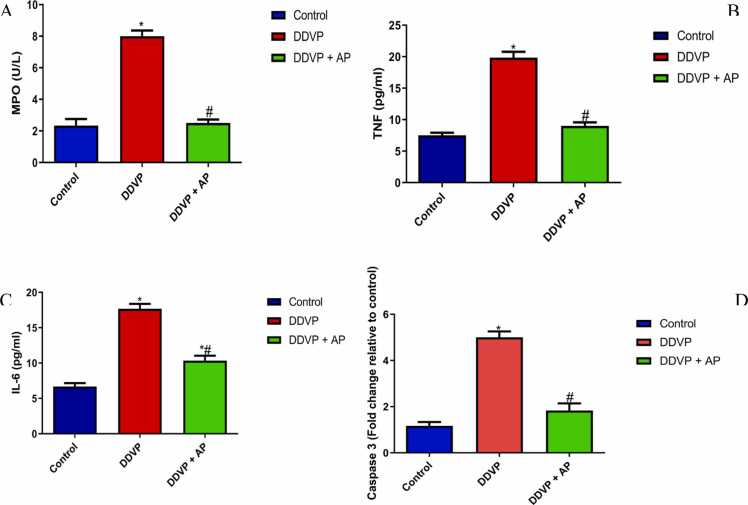
A- Myeloperoxidase (MPO); B-, Tumor necrosis factor-α (TNF-α); C- Interleukin-6 (IL-6); D- Caspase 3 in control, DDVP-treated and DDVP + AP rats. Each bar represents mean ± standard error of mean (S.E.M). *p < 0.05 vs control, ^#^p < 0.05 vs DDVP-treated. Dosage of DDVP= 98.54 g/m3; Dosage of AP= 500 mg/kg. DDVP: Dichlorvos; AP: *Andrographis paniculata*.

The effect of exposure to DDVP and treatment with *A. paniculata*are shown in Fig. 6. DDVP increased the levels of IL-6, TNF-α, MPO, and caspase-3 expression in contrast to rats used as controls. Treatment with *A. paniculata* at the dose of 500 mg/kg decreased the levels of IL-6, TNF-α, MPO, and caspase-3 expression.

#### 3.4.3 Immunohistochemistry

**Fig. 7:**
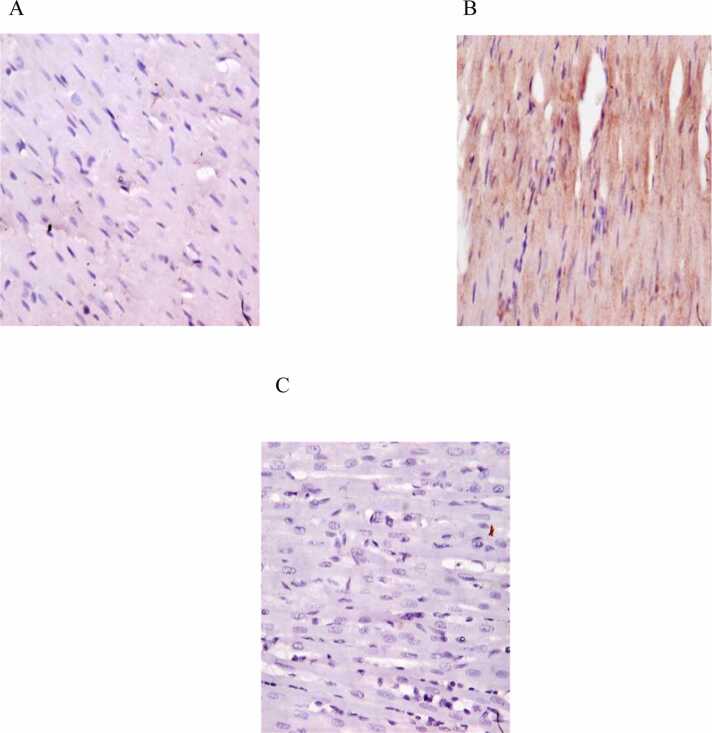
Photomicrograph (magnification x400) of immunohistochemistry assay of a heart tissue in control, DDVP-treated and DDVP+AP-treated rats. A- control showing no expression of Caspase 3in the cytoplasm of cardiac muscle; B- DDVP-treated showing moderate expression of Caspase 3in the cytoplasm of cardiac muscle; C- DDVP+AP-treated showing no expression of Caspase3 inthe cytoplasm of cardiac muscle. DDVP: Dichlorvos; AP: *Andrographis paniculata*.

**Fig. 8:**
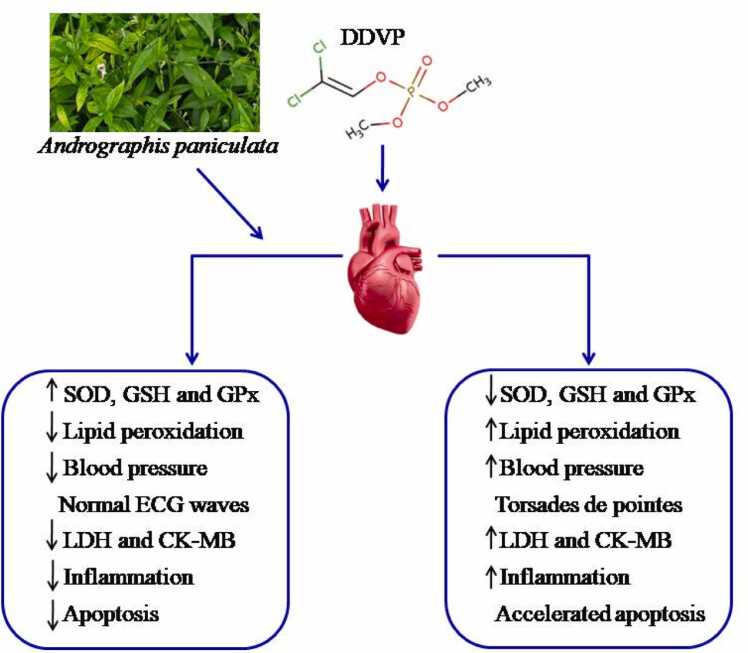
Graphical illustration of the effects of inhalation of DDVP and treatment with *Andrographispaniculata* on the heart.

Effects of exposure to DDVP and treatment with *A. paniculata* on cardiac histo-architecture are shown in Fig. 7. DDVP led to degradation of cardiac histo-architecture and elevated caspase-3 expression in contrast to rats used as controls. Treatment with *A. paniculata* at the dose of 500 mg/kg preserved the cardiac histo-architecture and decreased caspase-3 expression (Fig. 8).

#### 3.4.4 Phytochemical analysis

The results of the qualitative phytochemical tests are displayed in Table 1. Aqueous extract of *A. paniculata* contained alkaloids, tannin, saponin, terpenoids, flavonoids, glycoside and phytosterol.

**Table 1:** Phytochemical analysis of aqueous extract of *Andrographispaniculata*.

Phytochemicals	Presence
Alkaloids	++
Tannin	++
Saponin	+
Terpenoids	++
Flavonoids	++
Protein	-
Glycoside	+
Phytosterol	+

## 4 Discussion, conclusion, and recommendation

### 4.1 Discussion

People are now more exposed to environmental contaminants as a result of the ongoing usage of pesticides. These contaminants have negative health impacts that are both short-term and long-term. Pesticides' artificial ingredients could have long-term negative impacts on the ecosystem. Humans are also exposed to insecticides in dwellings, the entire toxicity profiles of which remain unknown. Dichlorvos (DDVP), a chemical that the WHO has designated as extremely hazardous and as class one (Class I) [66], builds up in people and has harmful impacts on many body organs [64].

The prevalence of cardiovascular diseases (CVD) is constantly rising despite the abundance of knowledge regarding them. This has created an immediate demand for new drug candidates that are safe, effective, and relatively affordable. *Andrographis paniculata*, a plant that has long been used to cure a variety of illnesses in traditional medicine, contains andrographolide, which has several medicinal benefits [61]. Its ability to block the expression of pro-inflammatory cytokines including interleukin-6 and tumor necrosis factor has demonstrated its strong anti-inflammatory properties [17]. The plant also reduces the production of nitric oxide, which contributes to oxidative stress and inflammation [3], [69]. The Indian and Chinese traditional medicine systems have a long history of using *A. paniculata* as a hepatostimulant and hepatoprotective agent, and it was shown to prevent injury and death of liver cells following exposure to concanavalin-A [56]. Extracts of *A. paniculata* prevented the high expression of liver transaminases in the serum, which is the hallmark of ethanol-induced hepatotoxicity. Aqueous and ethanol extract of *A. paniculata* is effective against several bacterial strains. The antimicrobial activities of *A. paniculata* have been confirmed in *Salmonella typhimurium, E. coli, Staphylococcus aureus, Streptococcus pneumonia*, and several others [24], [70].

This study investigated the effect of aqueous extract of *A. paniculata* on dichlorvos-induced toxicity on the heart of male Wistar rats. This was done by assessing the cardiac enzyme activities, inflammatory markers, antioxidant markers, electro-cardiac function, and cardiac apoptosis markers, which act as the biomarkers for heart function and integrity, respectively. In this study, the aqueous extract of *A. paniculata* has been shown to have a cardioprotective effect in DDVP-induced cardiotoxicity. This is demonstrated by an enhanced antioxidant defense system, a reduction in left ventricular dysfunction and hemodynamic impairment, inhibition of lipid peroxidation, and a prevention of myocyte injury marker enzyme leakage from the heart.

This study found that the administration of dichlorvos resulted in a significant alteration in cardiovascular variables, including increased systolic and diastolic blood pressure, mean arterial pressure (MAP), and a corresponding decrease in heart rate (HR) and pulse pressure (PP). These findings are consistent with previous research on the effects of dichlorvos-induced cardiac injury on the heart [49]. Because of problems with left ventricular contraction, DDVP may stimulate the adrenergic response, which could alter cardiovascular factors. It has been shown that DDVP induction significantly reduced arterial blood pressure (DBP, SBP, and MAP) in rats according to the result of Jun et al. [26]. Nevertheless, the DDVP-induced change in cardiovascular characteristics was reversed by the administration of an aqueous extract of A. paniculata. The current findings support the earlier conclusions of Shreesh et al. [57], who noted that extract from *Andrographis paniculata* protected against cardiac damage caused by isoproterenol [57]. Tannins may be the reason for *A. paniculata's* ability to control blood pressure [18].

The study evaluated various electrocardiac functions, including P-duration, PR-interval, QRS-complex, QT-interval, and R-amplitude. Rats that were induced with DDVP showed aberrant electrocardiac function, as shown by a substantial increase in QT-interval and a non-significant difference in P-duration, PR-interval, QRS-complex, and R-Amplitude when compared to control rats (as depicted in Fig. 3a-e). It is possible that DDVP predisposes to ventricular arrhythmia (torsades de pointes) and the accompanying seizure, as evidenced by the reported DDVP-induced increase in QT-interval [11]. This may be a factor in the study's findings that DDVP causes a rise in arterial blood pressure and a drop in heart rate and pulse pressure. Nevertheless, administration of *A. paniculata* aqueous extract resulted in a considerable reduction of increased QT-interval with a non-significant difference compared to dichlorvos-induced rats. The administration of an aqueous extract of A. paniculata reversed this alteration. This reveals the extract's cardioprotective effect, which is similar to the findings by Charan Sahoo et al., [15].

Among other enzymes, the heart contains large concentrations of lactate dehydrogenase and creatine kinase. However, if metabolic damage occurs, these enzymes are detected in the extracellular matrix [55]. A more sensitive marker in the early stages of myocardial ischemia is serum creatine kinase activity. In addition, peak increases in lactate dehydrogenase are generally correlated with the degree of cardiac tissue damage [16], [71]. When assessing the integrity of the cardiac apparatus in medication biotransformation and metabolism, blood levels of creatine kinase and lactate dehydrogenase could be employed [10]. The increased LDH and CK-MB infer that DDVP induces cardiac injury [62]. This is consistent with earlier research that showed the cardiotoxic effects of insecticides and pesticides containing pyrethroids [50], [51]. Aqueous extract of *A. paniculata* significantly ameliorated this effect [2].

Oxidative stress is the primary factor responsible for causing damage to the heart. Antioxidant enzymes are widely acknowledged as the first line of defense against oxidative stressors in order to safeguard cellular integrity and prevent the onset of numerous degenerative illnesses. The overproduction of free radicals during oxidative stress has a profound impact on the status of the enzymatic antioxidant enzymes, particularly SOD, catalase (CAT), and GPx, as well as the non-enzymatic antioxidant, GSH. This impact varies depending on the degree of disruptions in the normal redox state within the cells. Inflammatory diseases, alcoholism, smoking-related disorders, ischemia diseases, and many other conditions are significantly impacted by oxidative stress [20], [30], [44]. In the current investigation, the overall decline in the levels of enzymatic and non-enzymatic antioxidants in the heart homogenates of rats treated with dichlorvos suggested a net suppression of the tissue's total antioxidant capacity. This supports other reports [4], [50], [51]. Antioxidant status significantly reversed after treatment with *A. paniculata* aqueous extract, suggesting the extract's potential for amelioration. This finding implies that the plant extract has bioactive components that can scavenge free radicals by giving them hydrogen ions, so reducing their ability to cause cellular harm. This confirms the aqueous extract of *A. paniculata's* protective effect against oxidative stress brought on by dichlorvos exposure. The observed improvement in cardiac function seen in *Andrographis paniculata*-treated animals may be due to its antioxidant ability [2].

Lipid peroxidation is a sensitive marker of oxidative stress, an essential pathogenic event in myocardial necrosis induced by DDVP. Cardiac damage intensity is reflected by increased MDA level, a lipid peroxidation end product [28], [31]. This study showed a rise in MDA level in the group treated with DDVP which was attenuated by *Andrographis paniculata.* The reduced amount of MDA in heart tissues may have been brought about by the increased activity of antioxidant enzymes such as SOD and GPx. The extract's cardioprotective effects could have been caused by the efficient neutralization and scavenging of the free radicals that DDVP generated.

Inflammatory response is a key factor in developing various cardiovascular diseases, including myocardial infarction [26]. Patients with cardiovascular diseases exhibit increased expression and plasma concentrations of inflammatory markers and mediators [14], [54], [9]. It is a known fact that oxidative stress and increased production of reactive oxygen species (ROS) can cause inflammation and vice versa. Most often, oxidative stress amplifies a range of inflammatory cell signaling pathway [44]. The onset and progression of cardiac dysfunction are mostly dependent on inflammatory markers such TNF-α, IL-6, and MPO, which were significantly elevated after exposure to DDVP. The DDVP-induced increase in cardiac inflammatory markers may be due to DDVP-induced oxidative stress [6]. This may account for the increased cardiac injury markers and alterations in electro-cardiac function. The results are consistent with other studies showing that, despite DDVP's persistent binding and inhibition of acetylcholinesterase activity, the majority of its detrimental biological effects are mediated by oxidative stress [25], [65]. Administration of aqueous extract of *A. paniculata* significantly decreases cardiac inflammatory markers compared to dichlorvos-induced rats. The cardiac cytoprotection conferred by *A. paniculata* likely prevented inflammatory response in the cardiac tissue [6].

Cardiac apoptosis markers such as caspase 3 were assessed in this study. The increase in cardiac caspase 3 caused by DDVP infers that DDVP induces cardiac apoptosis [6]. DDVP likely triggered cardiac proton pump dysfunction with increased intracellular calcium ions, resulting in DNA fragmentation and cell death [6]. This may be because DDVP can cause inflammation and oxidative damage, which are known mediators of apoptosis [22], [47]. The administration of aqueous extract of *A. paniculata* likely abrogated caspase 3 by preventing cardiac oxidative injury and inflammatory response. The potential of *A. paniculata's* aqueous extract to protect against cardiotoxicity caused by DDVP could be explained by this.

### 4.2 Limitation

This study has some limitations and the primary constraint on this research is the challenge of determining the precise dosage of pesticide that humans are exposed to during a single application; the dosage used in this study cannot directly be related to human exposure. However, the behavioral pattern of humans following insecticide use, which means humans are exposed to a minute amount of insecticide in a single use, was considered, and the rats were exposed to a low dose of insecticide. This study draws strength from its methodology and a wide array of investigations. The study reported organophosphate's overall and specific effects on cardiovascular function, inflammation, oxidative stress, apoptosis, and cardiac architecture.

## 5 Conclusion

DDVP-induced cardiotoxicity via oxidative stress, inflammation, and apoptosis. Aqueous extract of *A. paniculata* confers cardioprotection against DDVP-induced cardiotoxicity by enhancing cardiac antioxidants. By restoring cardiac hemodynamic and contractile function, enhancing endogenous antioxidant defense, and inhibiting lipid peroxidation, the current findings highlight the therapeutic effects of *A. paniculata's* aqueous extract in an integrated approach. The study also showed that whole herb extract might be used for prophylaxis and treatment of CVD, as well as for excellent symptom relief. It is thought that the presence of phytochemicals, or tannins, or that the action of the plant as a whole inhibits lipid peroxidation or increases the activities of enzymatic antioxidants, is what makes the whole herb helpful against cardiovascular disease, inflammation, and apoptosis in the cardiac tissue.

## Recommendation

Further molecular studies demonstrating other possible mechanisms of DDVP-induced cardiac dysfunction are suggested. Studies exploring the beneficial effects of molecules other than *Andrographis paniculata* in DDVP-induced cardiotoxicity are also suggested.

## Abbreviation

CVD: Cardiovascular disease; WHO: World Health Organization; B.P: blood pressure; LDL: low-density lipoprotein cholesterol; HDL: high-density lipoprotein cholesterol; Ops: organophosphates; DDVP: Dichlorvos; ECG: Electrocardiography; MAP: mean arterial pressure; PP: pulse pressure; CK-MB: creatinine kinase isoenzyme; LDH; lactate dehydrogenase; SOD: superoxide dismutase; GPx: Glutathione peroxidase; MDA: malondialdehyde; GSH: reduced glutathione; MPO: Myeloperoxide; TNF-α: Tumor necrosis factor-alpha; IL-6: Interleukin-6; SBP: systolic blood pressure; DBP: diastolic blood pressure; CAT: catalase; ROS; reactive oxygen species; *A. paniculata: Andrographis paniculata*.

## Ethical approval and consent to participate

All necessary approval was obtained from the Faculty of Basic Medical Sciences, Ladoke Akintola University of Technology, Ogbomoso, Nigeria.

## Credit authorship contribution statement

**Saka Waidi Adeoye:** Writing – review & editing, Supervision, Methodology, Funding acquisition, Data curation, Conceptualization. **Olusanjo A. Ayandele:** Funding acquisition, Investigation, Methodology, Writing - original draft. **Oladapo Olusegun Oladipo:** Project administation, Writing - review & editing, final approval. **Olamilekan S. Adeshina:** Conceptualization, Funding acqusition, Writing - original draft, Project administration. **Busuyi David Kehinde:** Investigation, Validation and analysis

## Consent for publication

All authors agree to the final manuscript.

## Funding

No funding was obtained for this study.

## Declaration of Competing Interest

The authors declare that they have no known competing financial interests or personal relationships that could have appeared to influence the work reported in this paper.

## Data Availability

Data will be made available on request.
